# Payment mechanism for institutional births in Nepal

**DOI:** 10.1186/s13690-021-00680-7

**Published:** 2021-09-09

**Authors:** Ashish KC, Mats Målqvist, Amit Bhandari, Rejina Gurung, Omkar Basnet, Avinash K Sunny

**Affiliations:** 1 Department of Women’s and Children’s Health, Uppsala University, Dag Hammarskjölds väg 14B, Uppsala, Sweden; 2 Society of Public Health Physicians Nepal, Kathmandu, Nepal; 3 Golden Community, Lalitpur, Nepal

**Keywords:** Women, Childbirth, Out of pocket expenditure, Maternal incentive scheme, Nepal

## Abstract

**Background:**

Since the Millennium Development Goal era, there have been several efforts to increase institutional births using demand side financing. Since 2005, Government of Nepal has implemented Maternity Incentive Scheme (MIS) to reduce out of pocket expenditure (OOPE) for institutional birth. We aim to assess OOPE among women who had institutional births and coverage of MIS in Nepal.

**Method:**

We conducted a prospective cohort study in 12 hospitals of Nepal for a period of 18 months. All women who were admitted in the hospital for delivery and consented were enrolled into the study. Research nurses conducted pre-discharge interviews with women on costs paid for medical services and non-medical services. We analysed the out of pocket expenditure by mode of delivery, duration of stay and hospitals. We also analysed the coverage of maternal incentive scheme in these hospitals.

**Results:**

Among the women (n-21,697) reporting OOPE, the average expenditure per birth was 41.5 USD with 36 % attributing to transportation cost. The median OOPE was highest in Bheri hospital (60.3 USD) in comparison with other hospitals. The OOPE increased by 1.5 USD (1.2, 1.8) with each additional day stay in the hospital. There was a difference in the OOPE by mode of delivery, duration of hospital-stay and hospital of birth. The median OOPE was high among the caesarean birth with 43.3 USD in comparison with vaginal birth, 32.6 USD. The median OOPE was 44.7 USD, if the women stayed for 7 days and 33.5 USD if the women stayed for 24 h. The OOPE increased by 1.5 USD with each additional day of hospital stay after 24 h. The coverage of maternal incentive was 96.5 % among the women enrolled in the study.

**Conclusions:**

Families still make out of pocket expenditure for institutional birth with a large proportion attributed to hospital care. OOPE for institutional births varied by duration of stay and mode of birth. Given the near universal coverage of incentive scheme, there is a need to review the amount of re-imbursement done to women based on duration of stay and mode of birth.

**Supplementary Information:**

The online version contains supplementary material available at 10.1186/s13690-021-00680-7.

## Background

Sustainable Development Goal (SDG) 3 aims to achieve Universal Health Coverage (UHC) for essential health services by reducing the catastrophic expenditure on health [1]. One of the key barrier towards the achievement of UHC is out of pocket expenditure (OOPE) defined as direct payment for the cost of care [2, 3]. In order to mitigate financial barrier, there is a need to design effective evidence based interventions through a realistic financing strategies [4]. This is important as OOPE in many developing countries accounts for almost three-quarters or more of total expenditure on health [5–7]. Examples of financing scheme range from providing cash payments to mothers and families at the time of admission, voucher scheme during antenatal care, and reimbursing the cost of care at the health facilities [8–10]. Despite these efforts and investments by the government and international development agencies to address concerns over high OOPE, the inequity gap for utilizing health facilities during childbirth has further widened in the last decade [11].

‘Demand-side’ financing (DSF) has been defined as mechanism for transferring purchasing power to specified groups for the acquisition of defined goods or services [12]. In maternal health, DSF is used to reduce the financial cost of transportation, treatment and loss of earnings and have been done through either vouchers that can be exchanged for subsidized goods or specific services, or of short-term cash incentives or reimbursements that are linked to service use [13].Many countries have implemented health financing schemes (short payment scheme, voucher based system, conditional cash transfer and non-conditional cash transfer) for maternity care to promote institutional antenatal care and delivery [14]. These schemes are mainly targeted at reducing the economic burden of travel and treatment.

In 2019, GDP per capita in Nepal was 1071 USD in Nepal which ranks one of the lowest in South Asia [15]. Maternal financing scheme in Nepal was rationalized based on a study which showed that two thirds of the women do not reach to health facility due to financial barriers [16]. The Government of Nepal initiated in financing scheme to promote to promote institutional births and reduce financial barriers for women delivering at health facilities. This financial incentive scheme, Maternal incentive scheme (MIS) provided free childbirth services in 2005 [17]. The payment to women was gradual: NPR 1500 (13.8 USD) in mountain; NPR 1000 (9.2 USD) in hill; and NPR 500 (4.6 USD) in terai areas to reflect the higher costs in remoter areas [16, 17].

In January 2009, in addition to maternal incentive to women coming for childbirth, government of made all institutional birth free of cost across the country [18]. The new revised program was then called “Aama” Program. “Aama” program set a fixed reimbursement for various categories of childbirth and complication differentiated by size of facility [18].

In Nepal the institutional birth increased from that of 18 % in 2006 to 77.5 % in 2019 and MIS is one of the attributing factor to this increase [19–21]. There is very little evidence on coverage of maternal incentive scheme and out of pocket expenditure (medical or non-medical expenses) for childbirth. The medical related OOPE includes cost of admission, doctor, diagnostics, drugs and bed charge. The non-medical related OOPE includes cost of accommodation, travel and food. To provide evidence on the out of pocket expenditure for childbirth for informed programming of maternal incentive program, we aimed to assess out-of-pocket expenditure for institutional births and coverage of maternal incentive scheme in Nepal.

## Methods

An observational study nested within a large study to scale up quality improvement interventions in 12 public hospitals of Nepal was conducted between 1 and 2017 and the 17 October 2018 [22].

### Setting

All the hospitals included in this study are referral level public hospitals providing free delivery services and MIS, spread across the country, each with more than 1,000 deliveries per year. Four of the hospitals were high-volume (> 8,000 deliveries a year), four medium-volume (> 3,000 to 80,000 deliveries a year), and the remaining four low-volume (> 1,000 to 3000 deliveries a year) hospitals. Among the low volume hospitals, two (Nuwakot and Pyuthan) are located in hilly region and the other two (Bardiya and Nawalparasi) in terai region. All the high-volume hospitals (Koshi Zonal, Bharatpur, Lumbini Zonal, and Bheri Zonal) are located in terai region. Among the medium-volume hospitals, three (Western Regional, Rapti Sub-Regional and Mid-Western Regional) while one (Seti Zonal) hospital is located in terai region.

### Study participants

All the mothers who delivered in the hospitals during the study period were included in the study. Those mothers who did not consent or avail themselves for interview were excluded from the study.

***Socio-demographic characteristics*** included age of the mother categorized as < 20years, 20–35 years and ≥ 35 years; education categorized as Illiterate, Literate, Basic education, Secondary and above and Ethnicity categorized as Dalit, Janajati, Madhesi, Muslim, Chhetri/Brahmin-hill and Brahmin-Tarai.

***Obstetric variables*** included mode of delivery categorized as vaginal delivery, instrumental delivery and caesarian delivery; parity categorized as nullipara (never carried a pregnancy > 22 weeks), primipara (1 previous birth) and multipara (2–5 previous births); gestational age categorized as < 37 weeks, 37–41 weeks and ≥ 42 weeks; and birth weight categorized as < 2500 g, 2500–4000 g and ≥ 4000 g.

***Out of pocket expenditure*** was defined as expenses made for various services received at the hospital until discharge such as admission charge, bed charge, drugs and diagnostics and additional expenses made for transportation, accommodation for the caregiver and food.

***Maternal incentive*** was the cash payment the mothers received post-delivery on discharge for delivering at the hospital. The cash payment of NPR 500 (4.6 USD) was provided by the hospitals located in Terai region and NPR 1000 (9.2 USD) was provided by the hospitals located in hilly region as per the national incentive scheme.

### Data collection and management

Data were collected through a data surveillance system established in all hospitals. Data collectors extracted information on obstetric variables from the maternity registers and medical records using a data retrieval form. For information on socio-demographic and cost of care, a semi-structured interview was conducted by the data collectors with mothers at the time of discharge using an interview form. These completed forms were then assessed for completeness and accuracy by a data coordinator at the hospitals. Data were then entered into the database by the data entry and management team using the Census and Survey Processing System (CSPro).

### Statistical analysis

Data were exported into Statistical Package for Social Sciences (SPSS) version 23 for analysis. Descriptive statistics were presented with frequency, percentage, mean, standard deviation (SD), median and interquartile range (IQR). Kruskal Wallis test, Mann-Whitney test and Linear regression, and were used for comparing the cost of care across various variables between the groups. For Table 1, kruskal wallis test was used to assess the difference in OOPE by different sub-categories. For Table 2, multi-variable linear regression was used to assess the change in OOPE by duration of day and mode of delivery. Mann-Whitney test was done to compare the mean OOPE with or without maternal incentive. Missing data were excluded from the analyses.

**Table 1:** Out of pocket expenditure by mode of delivery, duration and hospital of birth (*n* = 21,697)

Out of pocket expenditure	Mean ± SD in USD	Median P50 (P25, P75) in USD	*p*-value*
**Mode of delivery** (***n*** = 21,602)			< 0.001
Vaginal delivery (n-15,311)	38.26 ± 25.82	32.6 (20.6–50.7)	
Instrumental delivery (n-841)	42.87 ± 24.27	37.8 (24.9–59.0)	
Caesarean delivery (n-5,450)	50.59 ± 30.22	43.3 (28.9–66.2)	
**Duration of stay**			< 0.001
1 to 2 days (n-14,124)	39.0 ± 25.5	33.5 (21.2–51.1)	
2 to 3 days (n-4,740)	45.9 ± 33.8	37.8 (25.0-60.3)	
4 to 7 days (n-1,719)	46.4± 28.3	41.8 (25.1–61.7)	
7 days or more (n-457)	53.2 ± 36.1	44.7 (26.5–68.7)	
**Hospital**			< 0.001
Surkhet Provincial hospital (n-2,034)	26.95 ± 19.62	19.8 (14.7–31.8)	
Bardiya hospital (n-89)	23.08 ± 9.90	20.5 (16.7–25.7)	
Bharatpur hospital (n-3,879)	36.60 ± 28.77	30.0 (22.3–40.7)	
Seti Provincial hospital (n-657)	41.70 ± 29.82	35.0 (20.3–52.5)	
Nuwakot hospital (n-464)	43.11 ± 29.81	35.3 (21.9–54.4)	
Koshi Provincial hospital (2,370)	31.18 ± 26.66	24.9 (16.3–38.4)	
Rapti hospital (2,560)	33.72 ± 29.46	23.9 (16.3–39.1)	
Prithivi Chandra hospital (159)	14.82 ± 5.99	13.5 (11.1–15.9)	
Lumbini Provincial hospital (8,922)	51.83 ± 23.40	47.4 (33.5–64.9)	
Bheri hospital (350)	57.33 ± 27.43	60.3 (41.5–65.4)	
Pythan hospital (213)	46.96 ± 29.84	42.4 (25.9–62.6)	

*Kruskal Wallis Test

**Table 2:** Linear regression to assess the change in OOPE by duration of stay and mode of birth

	OOPE in USD (95 % CI)	ß coefficient	*p*-value
Constant	30.2 (24.9, 35.4)		0.000
Per day OOPE	1.5 (1.2, 1.8)	0.093	0.000
Spontaneous vaginal birth	Reference		
Assisted vaginal birth	-0.2 (-5.7, 5.3)	-0.001	0.946
Caesarean birth	7.0 (1.7, 12.3)	0.111	0.01

Adjusting with hospitals

### Ethical approval and consent

Written informed consent was obtained from the mothers before inclusion in the study and confidentiality was maintained. The study was approved by Ethical Review Board of Nepal Health Research Council (reference number 26-2017).

## Results

**Fig. 1:**
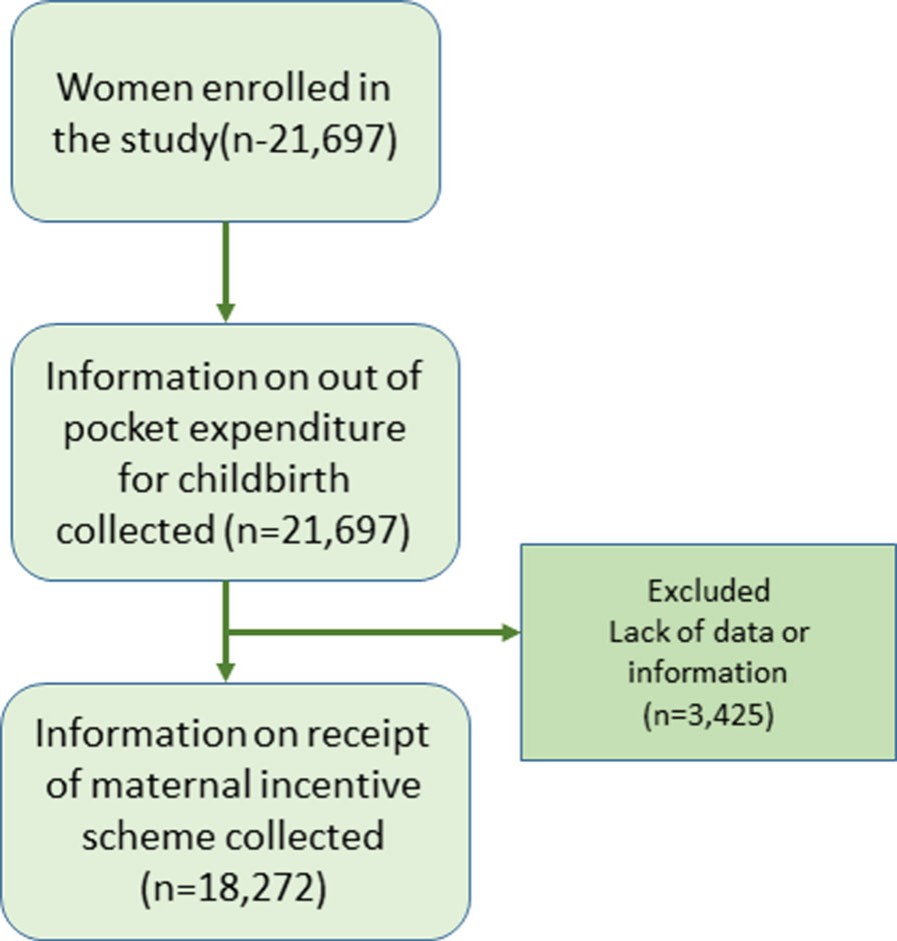
Study flow figure

**Fig. 2:**
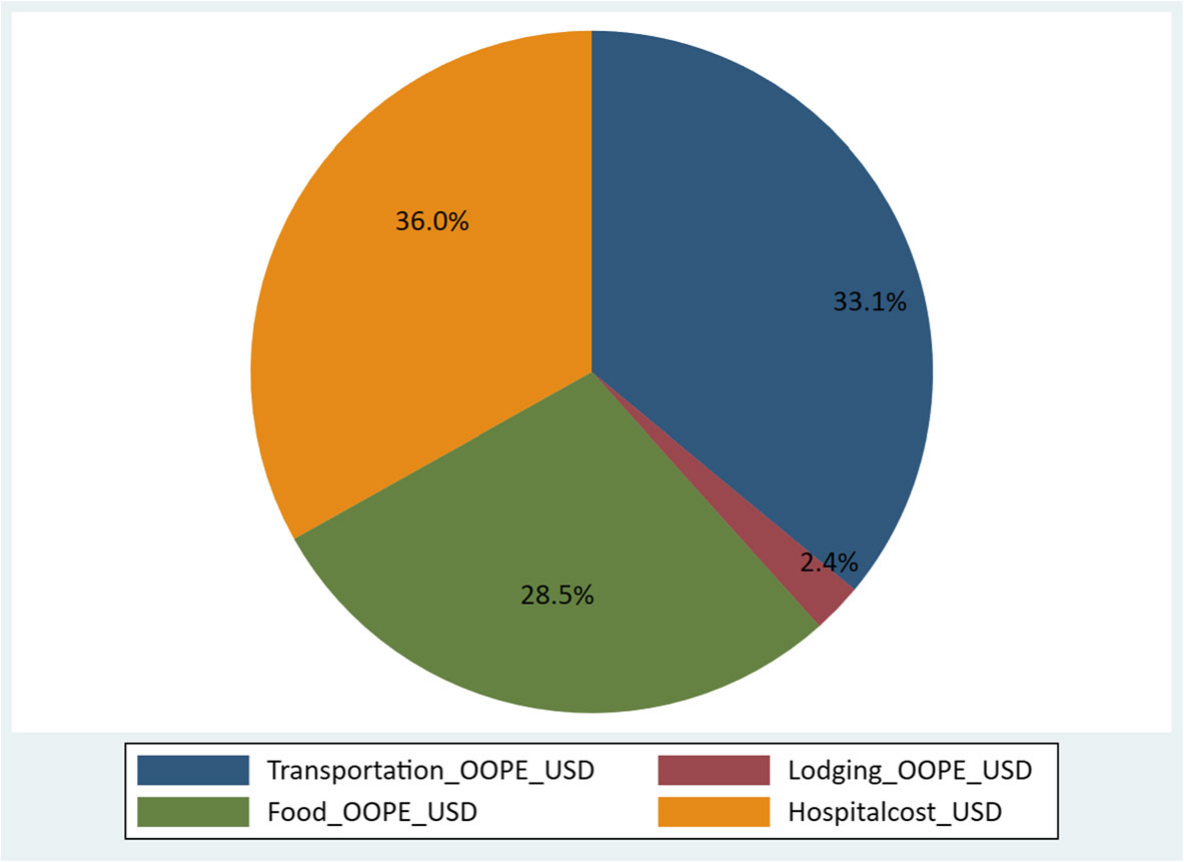
Distribution of Out of pocket expenditure mean 41.5 USD

Among the total women interviewed, 21,697 reported on out of pocket expenditure for childbirth (Fig. 1). Among the women interviewed the mean OOPE was USD 41.54. The Cost of hospital expenses accounted 36 % of total OOPE. The cost of transportation accounted 33.1 % and cost of food accounted 28.5 % of total OOPE (Fig. 2).

There was a difference in the OOPE by mode of delivery, duration of hospital stay and hospital of birth. The median OOPE was high among the caesarean birth with 43.3 USD in comparison with vaginal birth, 32.6 USD. The OOPE was 44.7 USD, if the women stayed for 7 days and 33.5 USD if the women stayed for 24 h. The OOPE was highest if the women delivered in Bheri hospital (60.3 USD) followed by Lumbini Provincial hospital (47.4 USD) (Table 1). The OOPE increased by 1.5 USD with each additional day of hospital stay after 24 h. The OOPE expenditure increased by 7.0 USD if the women had caesarean birth in comparison with vaginal birth (Table 2).

Among the women who agreed to interview, 96.5 % (95 % CI, 96.4–96.7) of them reported to have received the incentive. Among the hospitals, Koshi hospital had the lowest coverage (88.1 %, 95 % CI, 86.2–89.7) and Pythan hospital had the highest coverage (99.8 %, 95 % CI, 99.5–99.9). Women from madeshi, a relatively disadvantaged group had low coverage (93.1 %, 95 % CI, 91.4–94.4). Women from Brahmin-Tarai, relative advantaged had high coverage (98.4 %, 95 % CI, 97.6–99.0). The coverage was low among the caesarean birth (96.2 %, 95 % CI, 95.6–96.7) in relation with assisted vaginal birth (98.5 %, 95 % CI, 97.3–99.0) (Table 3).

**Table 3:** Coverage (%) of maternal incentive by background characteristics of women (*n* = 18,272)

Hospital	
Surkhet Provincial hospital	100 %
Bardiya hospital	100 %
Bharatpur hospital	93.4 % (92.3, 94.4)
Seti Provincial hospital	99.1 % (98.0, 99.6)
Nuwakot hospital	99.1 % (97.7, 99.7)
Koshi Provincial hospital	88.1 % (86.2, 89.7)
Rapti hospital	96.5 % (95.6, 97.2)
Prithivi Chandra hospital	100 %
Lumbini Provincial hospital	99.1 % (98.8, 99.3)
Bheri hospital	98.0 % (95.9, 99.0)
Pythan hospital	99.5 % (96.6, 99.9)
Ethnicity	
Dalit	97.4 % (96.7, 98.0)
Janajati	96.9 % (96.3, 97.3)
Madhesi	93.1 % (91.4, 94.4)
Muslim	97.2 % (95.2, 98.4)
Chhetri/Brahmin	98.1 % (97.7, 98.3)
Others	98.4 % (97.6, 99.0)
Maternal age	
< 20 years	97.4 % (96.4, 98.2)
20–35 years	97.3 % (97.1, 97.6)
> 35 years	97.0 % (95.0, 98.2)
Parity	
No previous birth	97.6 % (97.3, 97.9)
1 previous birth	96.9 % (96.5, 97.3)
2–5 previous births	97.3 % (96.5, 97.8)
Literacy	
No	97.4 % (97.1, 97.6)
Yes	96.1 % (94.2, 97.4)
Mode of birth	
Normal vaginal	97.7 % (97.4, 97.9)
Assisted vaginal	98.5 % (97.3, 99.1)
C-section	96.2 % (95.6, 96.7)

The OOPE for hospital expense was higher among those who did not receive maternal incentive than those who received it (17.4 vs. 13.2 USD, *p*-value < 0.001). The OOPE for transport was higher among those who did not receive maternal incentive than those who received it (16.7 vs. 15.1 USD, *p*-value < 0.001). There was no difference in the total OOPE for both the groups who received and did not receive maternal incentive (42.6 vs. 42.3, *p*-value-0.41) (Table 4).

**Table 4:** Distribution of OOPE by receipt of maternal incentive

	Mean±SD in USD	Received Maternal Incentive Scheme		***p***-value*
		No (*n* = 490), Mean±SD in USD	Yes (*n* = 17,783), Mean±SD in USD	
Transportation cost	15.0 (14.7, 15.2)	16.7 (14.8, 18.7)	15.1 (14.8, 15.4)	**<0.001**
Lodging cost	0.98 (0.97, 1.0)	0.95 (0.91, 0.98)	0.99 (0.97, 1.01)	0.689
Food cost	11.8 (11.6, 12.0)	7.3 (6.3, 8.2)	13.3 (13.1, 13.6)	**<0.001**
Hospital cost	13.8 (13.6, 13.9)	17.4 (16.3, 18.6)	13.2 (13.1, 13.3)	**<0.001**
Total	41.5 (41.2, 41.9)	42.4 (39.8, 45.0)	42.6 (42.2, 43.0)	0.41

^*^Mann Whitney test

## Discussion

The mean out of pocket expenditure was high in Nepal despite the near universal coverage of maternal incentive scheme. One third of the OOPE was due to hospital related expenses. OOPE was highest among the caesarean birth and for women staying for 7 days or more in the hospital, indicating that the OOPE varied with hospital related expense. The OOPE also varied by hospitals with women giving birth in Bheri hospital having the highest expense while women giving birth in Prithivi hospital having the lowest expense for care. The OOPE for hospital related expense was higher among women who did not receive maternal incentive scheme.

The coverage of maternal incentive to women varied by hospital with all most all women giving birth in Seti, Nuwakot, Lumbini and Pythan receiving the incentive. The coverage of MIS was less among women from madeshi ethnicity, relatively disadvantaged ethnic group than women from Chettri/Brahmin ethnicity, relatively advantaged ethnic group. Maternal Incentive Scheme was designed to reduce the financial barrier to come to the health facility. The incentive scheme coverage indicated the adequacy of implementation of the program in the hospitals. However, of the total OOPE, still one third of the cost is attributed to transportation. The hospital expense remains another large factor for OOPE for institutional birth.

A systematic review of DSF on payments to reduce cost of access institutional delivery or skilled attendance at birth in low and middle income countries indicates an increased use and reduction in maternal mortality as a result [14]. A study on the Janani Suraksha Yojana, maternal incentive scheme in India, demonstrated an increase in births in facilities in high-focus states compared to in non-high-focus states [23]. Janani Suraksha Yojana’s increased the births at healthcare facilities which provided t access to free 24-h care [24]. However, the study showed the quality of care did not improve on respectful care and clean birth practices [25].

The maternal health voucher scheme in Bangladesh implementation showed that in the project areas there the scheme was implemented odds of women seeking care for antenatal care increased two-fold and institutional birth three-fold [26]. In Cambodia, after the introduction of the voucher scheme and health equity fund, the institutional deliveries increased by almost 25 % in a span of 2 years [27].

There has been no study to assess the effectiveness of DSF on costs, cost-effectiveness and cost-utility of short-term payments [28]. The causal pathway of the possible effect of maternal incentive scheme is modified by contextual health system and social factors [29]. Maternal incentive scheme has been effective to improve health seeking behaviour considerably and health status to some extent [19]. The causal pathway of DSF’s functioning and effectiveness was not linear.

Evaluations spanning more than 15 years of implementation of maternal incentive programmes reveal a complex picture of experiences that reflect the importance of financial and other social, geographical and health systems factors as barriers to accessing care [30]. Careful design of these programmes as part of broader maternal and newborn health initiatives would need to take into account these barriers, the behaviours of staff, and the quality of care in health facilities. Research is still needed on the context of implementation of maternal incentive schemes, sustainability of financing and where they fit, or do not fit, with plans to achieve equitable universal health coverage.

There are some limitations in this study. This study did not assess the expenditure of maternal care after referral which may reflect the total OOPE. This study might have under-estimated the coverage of OOPE, as the maternal incentive is disbursed after discharge. Further, the women during the early postpartum period might not recall all the expense made. Some information could have resulted in social desirability bias as there is a wide range in terms of expenses reported. We assume that they may have sometimes reported information based on their interpretation which is socially relevant. Due to the near universal coverage of MIS, the number of women who did not receive MIS was 17,783 and who did not receive was 490, so the sample between the two groups was uneven. So, the mean value of distribution of OOPE between two groups is skewed. The OOPE among the women who did not had MIS was estimated based on the small sample size.

## Conclusions

More than 95 % of women delivering in hospitals received maternal incentive scheme and the coverage varies by maternal education and ethnicity. The OOPE is attributed to transportation cost and duration of hospital stay. There is a need to reassess the cost reimbursed by maternal incentive scheme in Aama program and revise the reimbursement such that the financial burden is reduced. The health service expenditure also indicates the effectiveness of the free health program.

## Supplementary Information

**Additional file 1: Additional Table. **Distribution of Out of pocket expenditure for sick newborn care by hospital

## Data Availability

The datasets used and/or analysed during the current study are available from the corresponding author on reasonable request.

## Abbreviations

aOR: Adjusted Odds Ratio
cOR: Crude Odds Ratio
CSPro: Census and Survey Processing System
MIS: Maternal Incentive Scheme
DSF: Demand Side Financing
SPSS: Statistical Package for Social Sciences
UHC: Universal Health Coverage
