# Supplementary material for: Payment mechanism for institutional births in Nepal
Source: Arch Public Health. 2021 Sep 9;79:163. doi: 10.1186/s13690-021-00680-7 (PMC8427872; doi:10.1186/s13690-021-00680-7)
Supplement: Supplementary file 1 — Additional file 1: Additional Table. Distribution of Out of pocket expenditure for sick newborn care by hospital [file 13690_2021_680_MOESM1_ESM.docx]

**Additional Table. Distribution of Out of pocket expenditure for sick newborn care by hospital**

|  | Transportation  Mean±SD in USD | Lodging  Mean±SD in USD | Food  Mean±SD in USD | Health Service  Mean±SD in USD | Total  Mean±SD in USD |
| --- | --- | --- | --- | --- | --- |
| Surkhet Provincial hospital | 8.7 (8.1, 9.4) | 0.88 (0.86, 0.90) | 8.6 (8.2, 9.0) | 8.7 (8.5, 8.9) | 26.9 (26.1, 27.8) |
| Bardiya hospital | 5.3 (4.1, 6.4) | 0.93 (0.84, 1.02) | 4.3 (3.1, 5.6) | 12.5 (11.5, 13.6) | 23.1 (21.0, 25.1) |
| Bharatpur hospital | 15.2 (14.4, 16.0) | 0.96 (0.93, 0.98) | 1.5 (1.3, 1.7) | 19.0 (18.6, 19.4) | 36.6 (35.7, 37.5) |
| Seti Provincial hospital | 9.9 (8.6, 11.3) | 2.1 (1.7, 2.4) | 14.9 (13.7, 16.1) | 14.8 (13.9, 15.7) | 41.7 (39.4, 44.0) |
| Nuwakot hospital | 15.8 (14.0, 17.6) | 1.0 (0.89, 1.12) | 14.2 (12.5, 16.0) | 12.1 (11.4, 12.7) | 43.1 (40.4, 45.8) |
| Koshi Provincial hospital | 14.3 (13.4, 15.1) | 0.94 (0.92, 0.95) | 4.4 (4.1, 4.7) | 11.6 (11.1, 12.0) | 31.2 (30.1, 32.2) |
| Rapti hospital | 14.5 (13.7, 15.4) | 0.99 (0.90, 1.1) | 5.0 (4.6, 5.4) | 13.2 (12.7, 13.6) | 33.7 (32.6, 34.9) |
| Prithivi Chandra hospital | 6.0 (5.2, 6.7) | 0.99 (0.89, 1.1) | 1.0 (0.96, 1.1) | 6.8 (6.2, 7.4) | 14.8 (13.9, 15.8) |
| Lumbini Provincial hospital | 17.3 (16.9, 17.7) | 0.96 (0.93, 0.98) | 20.0 (19.7, 20.3) | 13.6 (13.5, 13.7) | 51.8 (51.3, 52.3) |
| Bheri hospital | 10.7 (8.4, 13.0) | 0.91 (0.89, 0.92) | 30.8 (28.2, 33.4) | 15.0 (14.3, 15.7) | 57.3 (54.4, 60.2) |
| Pythan hospital | 17.0 (14.4, 19.6) | 0.92 (0.91, 0.93) | 20.0 (17.8, 22.1) | 9.1 (7.8, 10.4) | 47.0 (43.0, 51.0) |
